# Investigation of anti-cancer and migrastatic properties of novel curcumin derivatives on breast and ovarian cancer cell lines

**DOI:** 10.1186/s12906-019-2685-3

**Published:** 2019-10-21

**Authors:** Jinsha Koroth, Snehal Nirgude, Shweta Tiwari, Vidya Gopalakrishnan, Raghunandan Mahadeva, Sujeet Kumar, Subhas S. Karki, Bibha Choudhary

**Affiliations:** 1 Institute of Bioinformatics and Applied Biotechnology, Electronic City Phase 1, Bangalore, Karnataka 560100 India; 2 JK, SN, and VG are graduate students registered under Manipal Academy of Higher Education, Manipal, 576104 India; 3 Department of Biochemistry, Indian Institute of Science, Bangalore, 560012 India; 4 Department of Pharmaceutical Chemistry, KLE Academy of Higher Education and Research, KLE College of Pharmacy, Rajajinagar, Bangalore, KN India

**Keywords:** Cancer therapy, Curcumin derivatives, PA1, MDA-MB-231, Anti-cancer, Migrastatic

## Abstract

**Background:**

Curcumin is known for its multitude of medicinal properties, including anti-cancer and migrastatic activity. Efforts to overcome poor bioavailability, stability, and side effects associated with the higher dose of curcumin has led to the development of newer derivatives of curcumin. Thus, the focus of this study is to screen novel curcumin derivatives, namely ST03 and ST08, which have not been reported before, for their cytotoxicity and migrastatic property on cancer cells.

**Methods:**

Anti-cancer activity of ST03 and ST08 was carried out using standard cytotoxicity assays viz., LDH, MTT, and Trypan blue on both solid and liquid cancer types. Flow cytometric assays and western blotting was used to investigate the cell death mechanisms. Transwell migration assay was carried out to check for migrastatic properties of the compounds.

**Results:**

Both the compounds, ST03 and ST08, showed ~ 100 fold higher potency on liquid and solid tumour cell lines compared to its parent compound curcumin. They induced cytotoxicity by activating the intrinsic pathway of apoptosis in the breast (MDA-MB-231) and ovarian cancer cell lines (PA-1) bearing metastatic and stem cell properties, respectively. Moreover, ST08 also showed inhibition on breast cancer cell migration by inhibiting MMP1 (matrix metalloproteinase 1).

**Conclusion:**

Both ST03 and ST08 exhibit anti-cancer activity at nanomolar concentration. They induce cell death by activating the intrinsic pathway of apoptosis. Also, they inhibit migration of the cancer cells by inhibiting MMP1 in breast cancer cells.

## Background

Cancer is a disease characterized by abnormal proliferation of cells, which can evade anti-growth signals and invade other parts of the body [1]. Cancer cells thus affect the normal functioning of the organ, impairing the homeostasis of the body [2]. Various therapeutic strategies such as chemotherapy, hormone therapy, immunotherapy, radiation therapy, targeted therapy, gene therapy, have been utilized for the effective management of the disease [3, 4]. Despite having very advanced treatment, metastasis, and cancer relapse with resistance remain a significant challenge in cancer treatment. The resistance to treatment is due to a subpopulation of cells, called cancer stem cells [5, 6]. Recent evidence has shown that cancer stem cells undergo continuous mutations and are responsible for drug resistance and relapse. Targeting cancer stem cells is challenging, but identifying drugs which can work on stem cells can solve the relapse and resistance seen in most of the high-grade tumors [7].

Currently, cancer reports show that around 8.2 million people around the globe are suffering from the irreversible metastatic condition of malignant tumors with drug resistance [8]. Malignant tumors, characterized by its invasive and metastatic nature, increases the morbidity and mortality rates seen in cancer patients [9]. Cancer cell produces extracellular matrix-degrading enzymes (MMPs), which help metastasize [10]. Re-localization of malignant cells to distant organs leads to secondary tumor growth even after the complete removal of the tumor from the primary site. Available chemo-therapeutic drugs do not efficiently work on metastasized cancer cells [10]. Therefore, given the above facts, the challenge is to eliminate cancer-promoting cells (cancer stem cells) selectively.

**Fig. 1:**
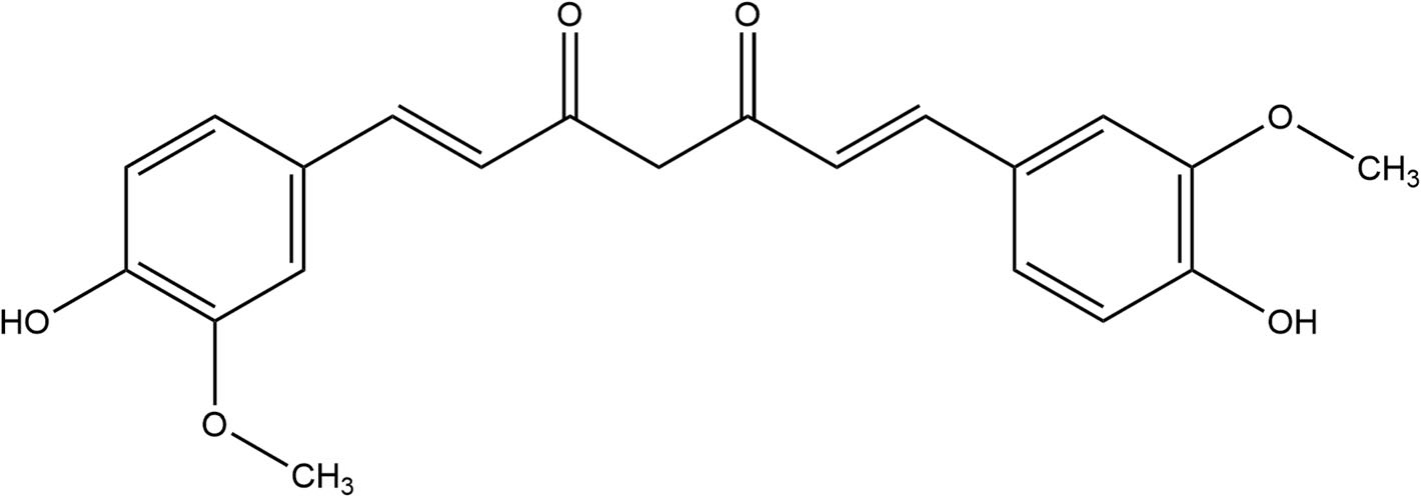
Curcumin chemical structure

Several natural compounds have shown to have anti-cancer activity. Among them Curcumin **(**Fig. 1**)** (1,7-bis (4-hydroxy 3-methoxyphenyl)-1,6-heptadione-3,5-dione or diferuloylmethane), has been shown to have anti-cancer on an array of cancer cells regardless of their origin [11–13]. Curcumin is derived from turmeric, and is known for it’s anti-inflammatory, anti-oxidant, anti-bacterial and anti-malarial properties [14, 15]. Studies have shown that curcumin can target cancer stem cells [16, 17] as well as inhibit cancer cell migration [8]. Curcumin has been shown to act on cancer stem cells of colorectal cancer, pancreatic cancer, breast cancer, brain cancer, and head and neck cancer [17]. Curcumin exhibits its anti-metastatic property by altering several signaling mechanisms, including inhibition of transcription factors, proteases, protein kinases, inflammatory cytokines, and their signaling pathways [10].

Although curcumin is known for its multitude of activities, it lacks stability and is not bioavailable in the in vivo system [18]. So to enhance the metabolic stability, various modifications were made onto the curcumin structures. DAPs (diarylidenyl-piperidone) is one of the well-studied groups of curcumin derivatives, which exhibited proliferation inhibition on multiple cancer cell lines such as colon, breast, ovarian epithelial cancer, etc. and multidrug resistance reverting property as well [19–23]. The multi-targeted effect of these compounds is documented to have more advantages than the single targeted ligands, as it can interfere with multiple signaling pathways and can have a pleiotropic effect [24–27]. S. Das et al.*,* have synthesized and demonstrated anti-cancer property of molecular dimers. They have conjugated two moieties of (3E, 5E)-3,5-dibenzylidenepiperidin-4-one pharmacophores via oxamide/propane diamide linkage. Their group has shown the anti-leukemic and anti-lymphoma activity of few 1,2-bis[(3E,5E)-3,5-dibenzylidene-4-oxo-1-piperidyl]ethane-1,2-dione derivatives [28–31]. The dimers of DAPs or 1,2-bis[(3E,5E)-3,5-dibenzylidene-4-oxo-1-piperidyl]ethane-1,2-dione attracted scientific attention to use as backbone structure due to its anti-cancer effect on various cancer types by activating the apoptotic pathway [29]. 1,2-bis[(3E,5E)-3,5-dibenzylidene-4-oxo-1-piperidyl]ethane-1,2-diones are thus considered as an excellent drug prototype for the development of novel compounds.

The dimers are relatively more stable than curcumin and also known to enhance the anticancer properties. Keeping the backbone of dimer constant, we synthesized two novel compounds, (ST03 (1,2-bis[(3E,5E)-3,5-bis[(2-chlorophenyl)methylene]-4-oxo-1-piperidyl]ethane-1,2-dione) and ST08 ([4-[(E)-[(5E)-1-[2-[(3E,5E)-3,5-bis[(4-hydroxyazonylphenyl)methylene]-4-oxo-1-piperidyl]-2-oxo-acetyl]-5-[(4-hydroxyazonylphenyl)methylene]-4-oxo-3-piperidylidene]methyl]phenyl] azinic acid)). We have checked anti-cancer activities of both the compounds on solid and liquid cancer cells. We have also investigated ST03 and ST08 induced cell death mechanism as well as their migrastatic property. We have carried out these studies on two major gynecological cancer types, breast, and ovarian cancer [32] using breast and ovarian cancer cell lines, respectively.

## Methods

### Chemistry

Silica gel plates were used for Thin Layer Chromatography by using toluene and ethyl acetate in 1:1 proportion. The IR spectra were recorded in KBr on a Jasco 430+ (Jasco, Japan); the ^1^H NMR spectra were recorded in CDCl_3_/DMSO on a Bruker (400 MHz), and J values were reported in Hertz (Hz). Mass spectra were recorded in triple quadrupole LCMS-6410 from Agilent technologies.

#### Procedure for synthesis of ST03 and ST08

##### ST03

Step 1.

Oxaloyl chloride (0.003 mol, 0.39 g) in DCE (5 mL) was added dropwise to a stirred suspension of a 3,5-bis (2-chlorobenzylidene)piperidin-4-one (0.006 mol) in DCE (20 mL) containing triethylamine (0.006 mol, 0.61 g) at 20 °C for a period of 30 min. The reaction was stirred at room temperature for 12 h. The solvent was removed under reduced pressure at 45 °C. An aqueous solution of potassium carbonate (25 mL, 5% w/v) was added to the crude mass and stirred for 2 h. The solid obtained was fifiltered, dried, and crystallized from 95% ethanol to yield the pure product.

Step 2:

The 2-chlorobenzaldehyde (26.71 mmol) was added dropwise to a suspension of 4-piperidone hydrochloride monohydrate (13.03 mmol) in acetic acid (35 mL). Dry hydrogen chloride gas was passed through this mixture until a clear solution was obtained. After stirring the reaction mixture at room temperature for 24 h, the precipitate was separated through filtration and added to a mixture of a saturated aqueous potassium carbonate solution (25% w/v, 25 mL) and acetone (25 mL); the resultant mixture was stirred for 0.5 h. The free base was collected, washed with water (50 mL), and dried. The compound was recrystallized from 95% ethanol to get the pure compound.

##### ST08

Step 1:

The 4-nitrobenzaldehyde (26.71 mmol) was added dropwise to a suspension of 4-piperidone hydrochloride monohydrate (13.03 mmol) in acetic acid (35 mL). Dry hydrogen chloride gas was passed through this mixture until a clear solution was obtained. After stirring the reaction mixture at room temperature for 24 h, the precipitate was separated through filtration and added to a mixture of a saturated aqueous potassium carbonate solution (25% w/v, 25 mL) and acetone (25 mL); the resultant mixture was stirred for 0.5 h. The free base was collected, washed with water (50 mL), and dried. The compound was recrystallized from 95% ethanol to get the pure compound.

Step 2:

Oxaloyl chloride (0.003 mol, 0.39 g) in DCE (1,2 Dichloroethane) (5 mL) was added dropwise to a stirred suspension of a 3,5-bis (4-nitrobenzylidene)piperidin-4-one (0.006 mol) in DCE (20 mL) containing triethylamine (0.006 mol, 0.61 g) at 20 °C for a period of 30 min. The reaction was stirred at room temperature for 12 h. The solvent was removed under reduced pressure at 45 °C. An aqueous solution of potassium carbonate (25 mL, 5% w/v) was added to the crude mass and stirred for 2 h. The solid obtained was filtered, dried, and crystallized from 95% ethanol to yield the pure product.

*ST-03:* Yield 45%, Rf 0.63, MP. 140–145 °C, IR (λ cm ^− 1^) 3061, 2975, 1642, 1440, 1260, 1044, 990. ^1^H NMR (δ): 7.99 (s, 2H), 7.94 (s, 2H), 7.53 (d, 2H, J = 9.2), 7.40–7.31 (m, 6H), 7.23 (d, 4H, J8.8), 7.18–7.14 (m, 2H), 7.05 (d, 2H, J = 9.2), 4.38 (s, 4H), 4.34 (s, 4H). MS (ESI) m/z: 742.53 (742.47).

*ST-08*: Yield 40%, Rf 0.55, MP. 180–182 °C, Nitro derivative IR (λ cm ^− 1^) 3075, 2932, 2851, 1666, 1599, 1520, 1346, 1263, 987. ^1^H NMR (δ): 8.33–8.25(m, 8H, ar), 7.77(s, 2H), 7.70 (d, 4H, J = 8.8 Hz), 7.63 (d, 4H, J = 8.8 Hz), 7.50 (s, 2H), 4.54 (s, 4 HO, 4.48 (s, 4H)). MS (ESI) m/z: 781.58 (784.68).

### Cell lines and culture

In order to investigate the anti-cancer activity of ST03 and ST08 on solid and liquid type of cancers, they were tested on multiple human cancer cell lines. Human cancer cell lines such as PA1 (ovarian teratocarcinoma cell line), MCF7 (breast adenocarcinoma cell line), MDA-MB-231(breast adenocarcinoma cell line), CEM (T acute lymphoblastic leukemia cell line), K562 (B chronic myelogenous leukemia cell line), A431 (epidermoid carcinoma cell line), HeLa (cervical adenocarcinoma cell line) and 293 T (embryonic kidney cell line) cells were purchased from NCCS, Pune, India. A2780 (ovarian endometrioid adenocarcinoma) was purchased from ATCC and Nalm6 (B cell precursor leukaemia cell line) was a kind gift from SCR lab, IISc, Bangalore, India). CEM, K562, Nalm6 cell lines were grown in RPMI-1640 (Lonza). PA1, MCF7, HeLa, A431 were grown in MEM and MDA-MB-231, 293 T cell lines were grown in DMEM media (Lonza). All cell lines were supplemented with 10% Fetal bovine serum and 1X antibiotic-antimycotic (GIBCO, Thermo Fisher Scientific, US) and maintained at 37 °C in a humidified incubator with 5% CO_2_ supply. Cytotoxicity of these compounds on normal cells was examined using peripheral blood mononuclear cells (a kind gift from SCR lab, IISc, Bangalore, India) and 293 T cells.

### MTT assay

Cytotoxicity exerted by ST compounds on cell lines were assessed by doing 3-(4,5-dimethylthiazol-2-yl)-2,5-diphenyltetrazolium bromide assay (MTT assay) [33]. Cells were seeded (5000 cells/well) in 96-well plate in triplicates, incubated for 24 h, and treated with respective ST compounds from 1 nM to 1000 nM. Curcumin was used for comparing the potency with its derivatives. Curcumin was added in the range of 1 uM to 100 uM concentrations to compare the cytotoxicity with its derivatives. After 48 and 72 h incubation, 10 μl of MTT (5 mg/mL) reagent was added to each well to a final concentration of 0.25 mg/mL and incubated till the colour developed. Following colour development, the reaction was stopped by adding stopping solution (50% N, N-Dimethylformamide) (Sigma–Aldrich, USA), 10% Sodium dodecyl sulfate, (MP Biomedicals, USA) and kept for 2 h incubation at 37^°^ C for complete solubilization of formazan crystal. Absorbance was measured at 570 nm on a 96 well plate reader (Tecan infinite 200 ELISA plate reader, Tecan Trading AG, Switzerland). Absorbance from culture medium without cells was considered as blank and was subtracted. Cells treated with an appropriate concentration of DMSO was used as vehicle control as the compounds were dissolved in DMSO. The 50% inhibition concentration of the drugs (IC_50_ values) were calculated from the 48 h treatment readings using GraphPad Prism 7 software. Percentage of viable cells in each treatment concentrations were calculated as a ratio of sample OD to the control OD.

### LDH assay

Lactate dehydrogenase (LDH) release is an indication of cell injury. This assay quantitatively measures the stable LDH in the cytosol. To perform this assay, 5000 cells/well were seeded in 96-well plate in triplicates and were treated with ST compounds (1 nM to 1000 nM). After 48 and 72 h of treatment, each well of 96-well plate was washed to remove FBS content and the cells were lysed using 0.5% Triton-X-100 prepared in 1X Phosphate buffer saline. This lysate was mixed with LDH assay reagents, described by OPS Diagnostics LLC, P.O. Box 348, Lebanon, NJ 08833 USA. The absorbance of the orange-red colored formazan product was measured at 490 nm using Tecan infinite 200 ELISA plate reader (Tecan Trading AG, Switzerland).

### Trypan blue exclusion assay

The cytotoxic effect of ST compounds on the viability of cancer cell lines was determined by Trypan blue exclusion assay [33]. The cells were seeded in 6 well culture plate at a density of 75,000 cells/mL and incubated for 24 h and treated with different concentrations of ST compounds (1 nM to 1000 nM). Cells were collected at 48 h and 72 h time points and resuspended in 0.4% of trypan blue (Sigma–Aldrich, USA). The number of viable cells was counted using hemocytometer. Percentage of viable cells in each treatment concentrations were calculated as a ratio of sample cell count to the control cell count. The IC_50_ values (50% inhibition concentration of the drugs) were calculated from the 48 h data.

### Phosphatidylserine externalization assay using AnnexinV-FITC/PI

In order to understand the mode of cell death (apoptosis/necrosis) induced by ST compounds in ovarian and breast cancer cells, annexin V-FITC/PI staining was carried out. Cells grown in 6-well plate with a cell density of 75,000 cells/mL were treated with ST compounds for 48 h. Cells were trypsinized, washed with ice-cold 1X Phosphate buffer saline and resuspended in 1X annexin binding buffer containing annexin V-FITC antibody (Biolegend, San Diego, CA) for 15 min in the dark on ice. PI (propidium iodide) was added (3.3 μg/mL) just before acquiring the samples. Cells incubated in 3% paraformaldehyde was used as a positive control. A total of 10,000 events were acquired for each sample using Beckman coulter Gallios flow cytometer (Beckman Coulter, Miami, FL)**.**

### Western blot analysis

Western blot analysis was carried out to examine the expression of proteins involved in the apoptotic pathway. To perform this assay, 75,000 cells/mL were seeded and treated with ST compounds (10 nM, 20 nM, 40 nM, 60 nM, 80 nM) for 48 h and whole cell lysate was prepared using RIPA buffer (25 mM Tris-Cl pH 7.6, 150 mM Sodium chloride, 1% NP-40, 1% Sodium deoxycholate, 1% Sodium Dodecyl Sulphate, 1 mM Phenylmethylsulphonyl fluoride, 1 mM Sodium orthovanadate). Crude cell lysates (30–40 μg) were electrophoresed on SDS-PAGE (Sodium dodecyl sulfate-polyacrylamide gel electrophoresis) and were transferred on to Polyvinylidene fluoride membrane (Millipore, USA) to probe with respective antibodies. The primary antibodies against caspase 9, cleaved caspase 9, caspase 3, cleaved caspase 3, caspase 8 and Horseradish peroxidase-labeled secondary anti-rabbit antibodies were purchased from Cell Signalling Technology, Beverly, MA. Anti-tubulin, Anti-MMP1 and its secondary mouse antibody were purchased from Santa-Cruz Biotechnology, Santa Cruz, CA. The membrane was probed with appropriate antibodies and was developed using chemiluminescence reagent (Clarity Western ECL blotting substrate, Biorad). The blot image was captured by using a Syngene G: Box gel doc system. Protein band image quantification was done using GelQuant. Net, Biochem Lab solutions.

### Transwell migration assay

Transwell assay was performed by seeding 75,000 cells/mL in a 6-well plate and were allowed to grow for 24 h. Forty and eighty nM ST08 treatment was done for 24 h. Permeable migration chambers were purchased from Corning Inc. (24-well insert; pore size, 8 μm) and were coated with 75 μL of matrigel and incubated at 37 °C for 24 h for settling. Fifty thousand cells/ millilitre treated cells were suspended in 200 μL media without FBS and added into the top chamber. Migration was allowed to occur for 5 h in a CO_2_ incubator. Then cells were fixed with 4% paraformaldehyde and stained with 2% crystal violet. Cells that did not migrate to lower compartment were cleaned using a cotton swab. Each insert was imaged in for five random fields at 10X magnification and analysis was done using NIH ImageJ software. Two independent experiments were carried out in duplicates.

### Statistical analysis

Data from 3 different biological replicates were collected and values are expressed as Mean ± SE in bar graphs. One-way or Two-way ANOVA followed by Tukey’s multiple comparison test was carried out and significance is represented as **** (*p*-value ≤0.0001), *** (*p*-value ≤0.001), ** (*p*-value ≤0.01), * (*p*-value ≤0.05). Statistical analysis was done using GraphPad Prism 7 tool.

## Results

### Characterization of ST03 and ST08

ST03 (1,2-bis[(3E,5E)-3,5-bis[(2-chlorophenyl)methylene]-4-oxo-1-piperidyl]ethane-1,2-dione) and ST08 ([4-[(E)-[(5E)-1-[2-[(3E,5E)-3,5-bis[(4-hydroxyazonylphenyl)methylene]-4-oxo-1-piperidyl]-2-oxo-acetyl]-5-[(4-hydroxyazonylphenyl)methylene]-4-oxo-3-piperidylidene]methyl]phenyl] azinic acid) were prepared based on the procedure given above. The structures of the synthesized compounds were confirmed by IR, NMR, and Mass spectrometry. The vibration of C-H bonds was observed between 3075 and 3003 cm^− 1^ whereas for aliphatic C-H bonds observed between 2946 and 2840 cm^− 1^, and for C=O bonds observed between 1666 and 1640 cm^− 1^. In ^1^H NMR, all the synthesized compounds showed prominent signals for aromatic and olefinic protons between δ 7.99–6.64 ppm. The structures of all the compounds were ensured by mass spectrometry. The detailed characterization results of the compounds are provided in the supplementary section (Additional file 1: Figures S1 and S2).

### ST03 and ST08 exert cytotoxicity on cancer cell lines with least effect on normal cells

ST03 and ST08 compounds were examined for its cytotoxicity on a) leukemic cell lines: CEM, K562, Nalm6 b) ovarian cancer cell lines: PA1 and A2780 c) Breast cancer cell lines: MCF-7, MDA-MB-231 d) 293 T (normal kidney cell line) e) A431 (skin cancer cell line) f) HeLa (cervical cancer cell line) and also on g) PBMC (peripheral blood mononuclear cells) using MTT, LDH, and Trypan blue exclusion assays. In the pilot screening experiment, all cells were treated with five different concentrations (1 nM to1 μM) of compounds (ST03 and ST08) for 48 and 72 h. Both the compound showed cytotoxicity at sub-micromolar concentrations. DMSO was used as the vehicle control. IC_50_ values of these compounds were calculated using all three methods and is listed in (Table 1 and 2).

**Table 1:**
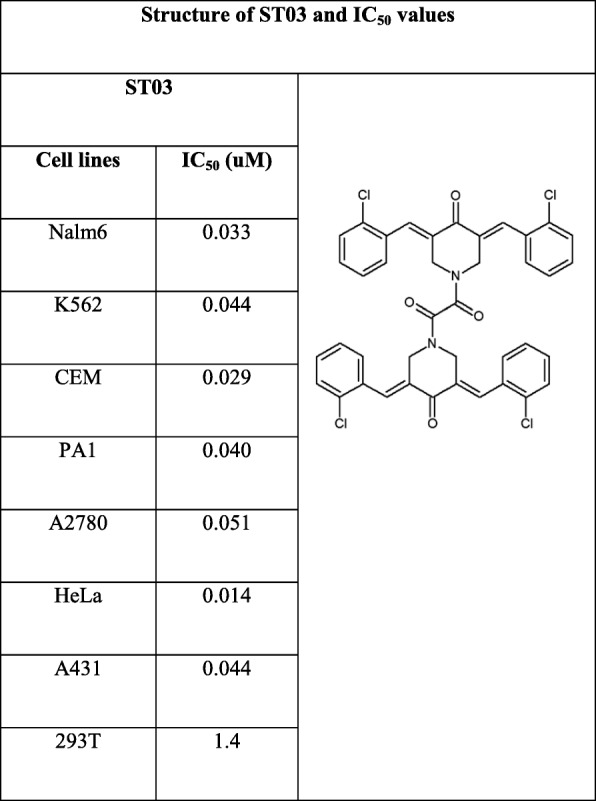
Structure of ST03 and IC_50_ values. IC_50_ values were calculated from the average IC_50_s of all cytotoxicity assays conducted on particular cell line at 48 h, and the results are summarized in micromolar concentrations

**Table 2:**
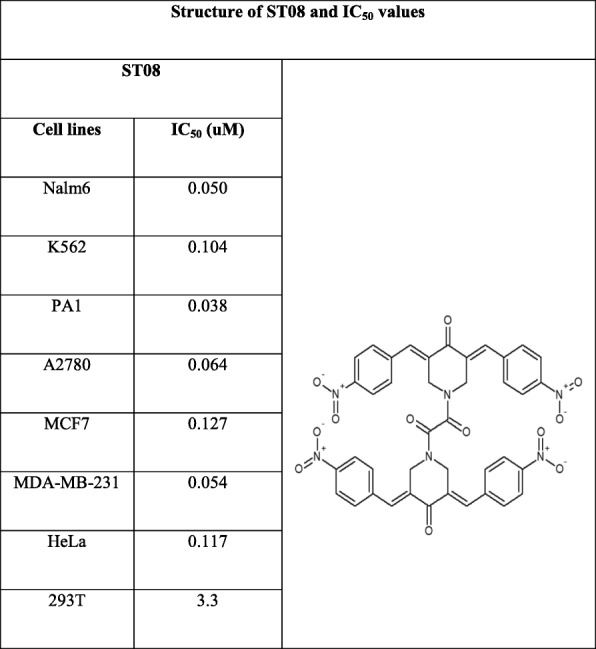
Structure of ST08 and IC_50_ values. IC_50_ values were calculated from the average IC_50_s of all cytotoxicity assays conducted on particular cell line at 48 h, and the results are summarized in micromolar concentrations

Curcumin showed 50% cytotoxicity in the range of 7–10 μM, ST compounds were in 0.030–0.080 μM range. Both, ST03 and ST08, showed ~100x higher potency than the parent compound curcumin. (Tables 1 and 2, Fig. 3c, Additional file 1: Table S1).

**Fig. 2:**
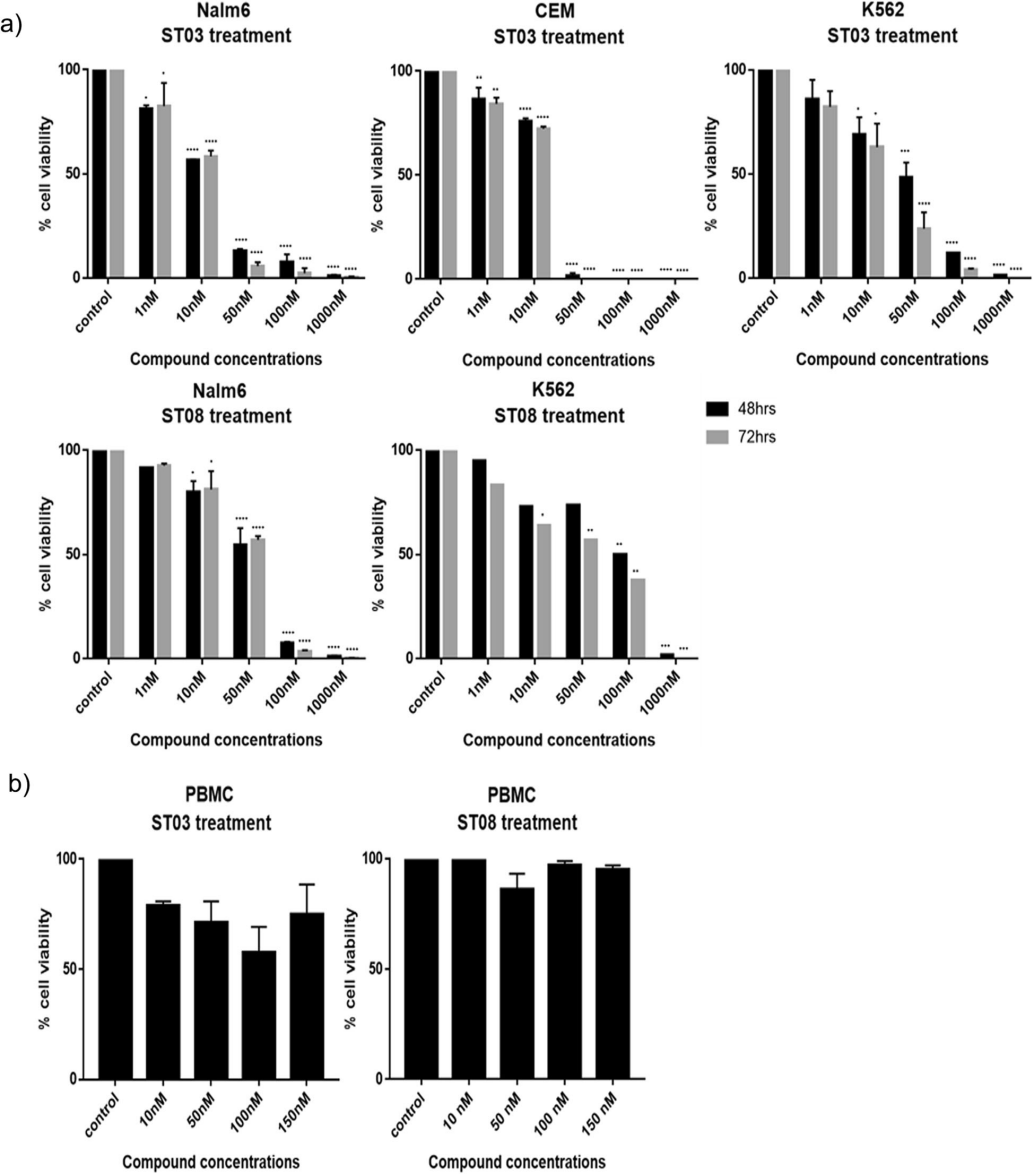
Evaluation of effect of ST03 and ST08 on cancer cell lines and PBMCs by Trypan Blue exclusion assay. **a** Viable cells after ST03 (Nalm6, CEM, K562) and ST08 (Nalm6, K562) treatment **b** Viable PBMC cell count after ST03 and ST08 treatments. Each experiment was repeated for a minimum of 3 times and plotted as bar graphs with error bars. Two-way ANOVA was conducted using Graph pad prism 7 tool and the *p* value was calculated between control and ST compound treated samples, where, *: *p* value < 0.05, **: *p* value < 0.005, ***: *p* value < 0.0001, ****: *p* value < 0.00001

Since ST compounds exhibited cytotoxicity at nanomolar concentrations, the next series of cytotoxicity assays were carried out in a range of 1 nM to 1000 nM to calculate IC_50_. Initial experiments were carried out on leukemic cell lines (CEM, K562, and Nalm6) using trypan blue assay. Cells were treated with an equivalent amount of DMSO as vehicle control. Dose-Dependent induction of cell death was seen in ST03, and ST08 treated cells. The lowest concentration (1 nM) was found to be least effective on all the cell lines tested (Fig. 2a). Nonetheless, at concentrations such as at ~ 30–50 nM onwards, we observed effective cytotoxicity, and 1000 nM showed maximum inhibition at 48 h and 72 h (Fig. 2a). No cytotoxicity was observed with the vehicle control, DMSO (0.1%). From these results, it was evident that ST03 and ST08 are toxic to human leukemic cell lines, and ST03 showed relatively higher potency than ST08. Peripheral blood mononuclear cells were used as normal cells to test cytotoxicity. The cells were treated with ST03 and ST08 for 48 h and cell viability assessed. No cytotoxicity was observed at doses three times more (150 nM) than the effective dose on cancer cell lines (~ 30–50 nM).

**Fig. 3:**
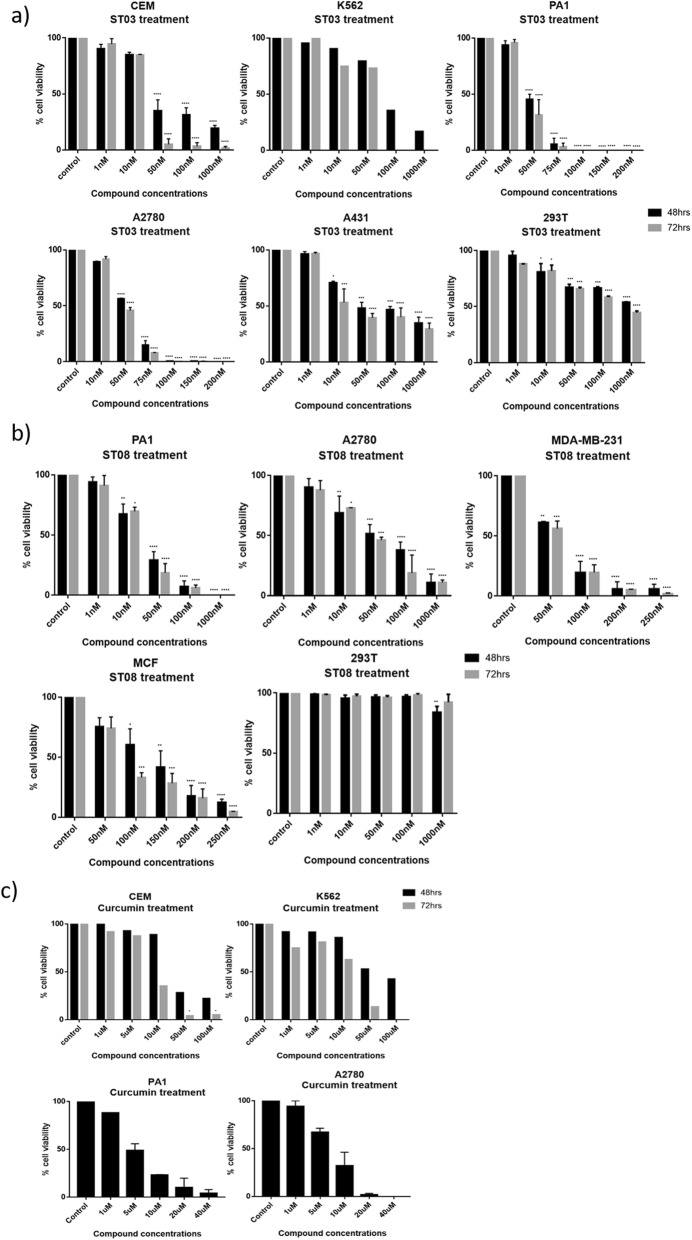
Evaluation of the effect of ST03, ST08 and curcumin compounds on cell lines by MTT assay. **a** ST03 treatment on PA1, A2780, K562, CEM,A431 and 293 T. **b** ST08 treatment on PA1, A2780, MDA-MB-231, MCF-7 and 293 T. **c** Curcumin treatment on PA1, A2780, CEM and K562. Each experiment was repeated for a minimum of 3 times and plotted as bar graphs with error bars. Two-way ANOVA was conducted using Graph pad prism 7 tool and the p value was calculated between control and compound treated groups, where,*: *p* value < 0.05, **: *p* value < 0.005, ***: *p* value < 0.0001, ****: *p* value < 0.00001

Additionally, MTT assay was also performed to check cytotoxicity in both liquid (CEM, K562) and solid cancer cell lines (PA1, A2780, A431, MDA-MB-231, MCF7, and 293 T (Fig. 3) following treatment with ST03 and ST08 for 48 h and 72 h. The reduction of MTT by live-cell mitochondria was considered directly proportional to cell proliferation. A dose-dependent (Fig. 3), decrease in the cell viability was observed. Both the drugs showed IC50 in the range of 30-50 nM in all the cell lines tested. Keeping our interest in developing drugs against metastatic ovarian and breast cancer, we tested, ST03, and ST08 induced cell death in (PA-1, ovarian teratocarcinoma vs. A2780, epithelial cancer). ST03 showed IC50 of 41 nM in PA1 vs. 54 nM in A2780 (Fig. 3a). Whereas, ST08 showed higher inhibition on PA1 (~ 38 nM) among the other cell lines tested (Fig. 3b). Among the breast cancer cell line MCF-7 (epithelial) and MDA-MB-231 (metastatic), MDA-MB-231(54 nM) showed better cytotoxicity than MCF-7 (127 nM) upon ST08 treatment. ST03, and ST08 showed the least toxic effect on normal kidney cell lines (293 T) as expected. These results again confirmed that both the compounds are cytotoxic to cancer cell lines rather than on normal cells. We also performed MTT with curcumin and found its IC_50_ in most cell lines to be in the range of ~ 10 uM-100 uM which is approximately 100 times more than ST03 and ST08 (Fig. 3c, Additional file 1: Table S1).

Also, LDH (lactate dehydrogenase) assay was conducted to confirm the above-observed results by examining the cell damage caused by ST03 and ST08 treatment. LDH is a cytosolic enzyme present in most of the eukaryotic cells. When the plasma membrane integrity is lost during the cell death process, it leaks out of the cell [34]. LDH reduces NAD^+^ to NADH with the help of lactate, and reduced NADH catalyses the formation of red formazan from INT with the help of PMS in the assay system. Thus, the presence of cytosolic LDH leads to the formation of red-coloured formazan, which directly correlates to the live cells present in the assay system.

**Fig. 4:**
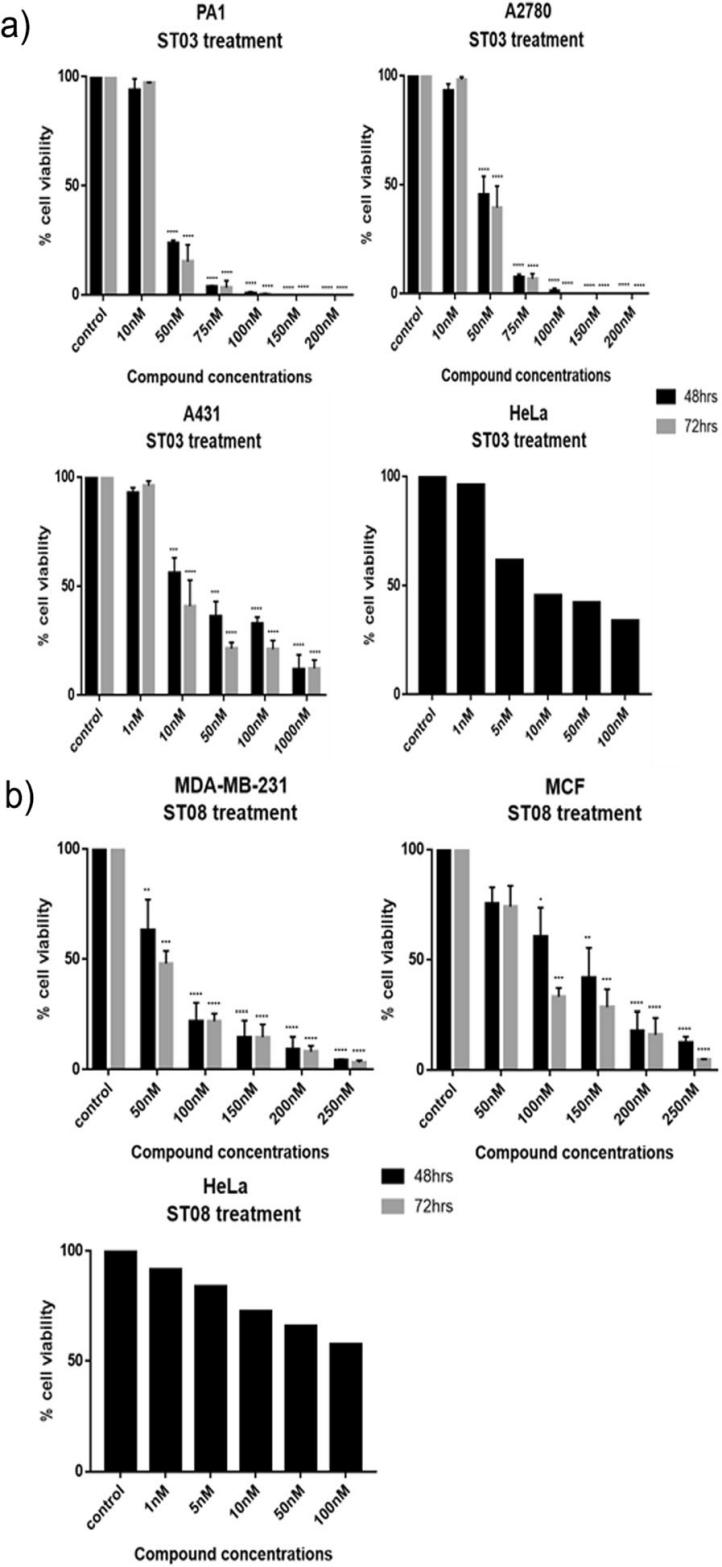
Examination of the effect of ST03 and ST08 compounds on cancer cell lines by LDH assay. **a** ST03 treatment on PA1, A2780, A431, HeLa and **b** ST08 treatment on MDA-MB-231, MCF-7, HeLa. Each experiment was repeated for a minimum of 3 times and plotted as bar graphs with error bars. Two-way ANOVA was conducted using Graph pad prism 7 tool and the p value was calculated between control and ST treated groups, where,*: *p* value < 0.05, **: *p* value < 0.005, ***: *p* value < 0.0001, ****: *p* value < 0.00001

PA1, A2780, A431, HeLa (Fig. 4a) cells were treated with ST03 and MDA-MB-231, MCF7 and HeLa (Fig. 4b) were treated with ST08 for 48 and 72 h and checked for cytoplasmic LDH content in each treatment (Fig. 4). LDH assay showed better sensitivity than MTT upon ST03 and ST08 treatment on cancer cell lines. However, the inhibitory concentration of both the compounds was in the same range. Here, among the ovarian cell lines tested, ST03 showed a better effect on PA1 cells (~ 39 nM) which is an ovarian teratocarcinoma cell line than A2780 (~ 49 nM) epithelial cancer of ovary (Fig. 4a). On the other hand, ST08 was more effective on the MDA-MB-231 cell line (~ 53 nM) than MCF7, the other breast cancer cell line tested. Although MCF7 is also a breast cancer cell type, it showed relatively less effectiveness (~ 125 nM) when compared to the triple-negative breast cancer cell type, MDA-MB-231 (Fig. 4b).

### ST03 and ST08 induced apoptosis in ovarian and breast cancer cell lines

**Fig. 5:**
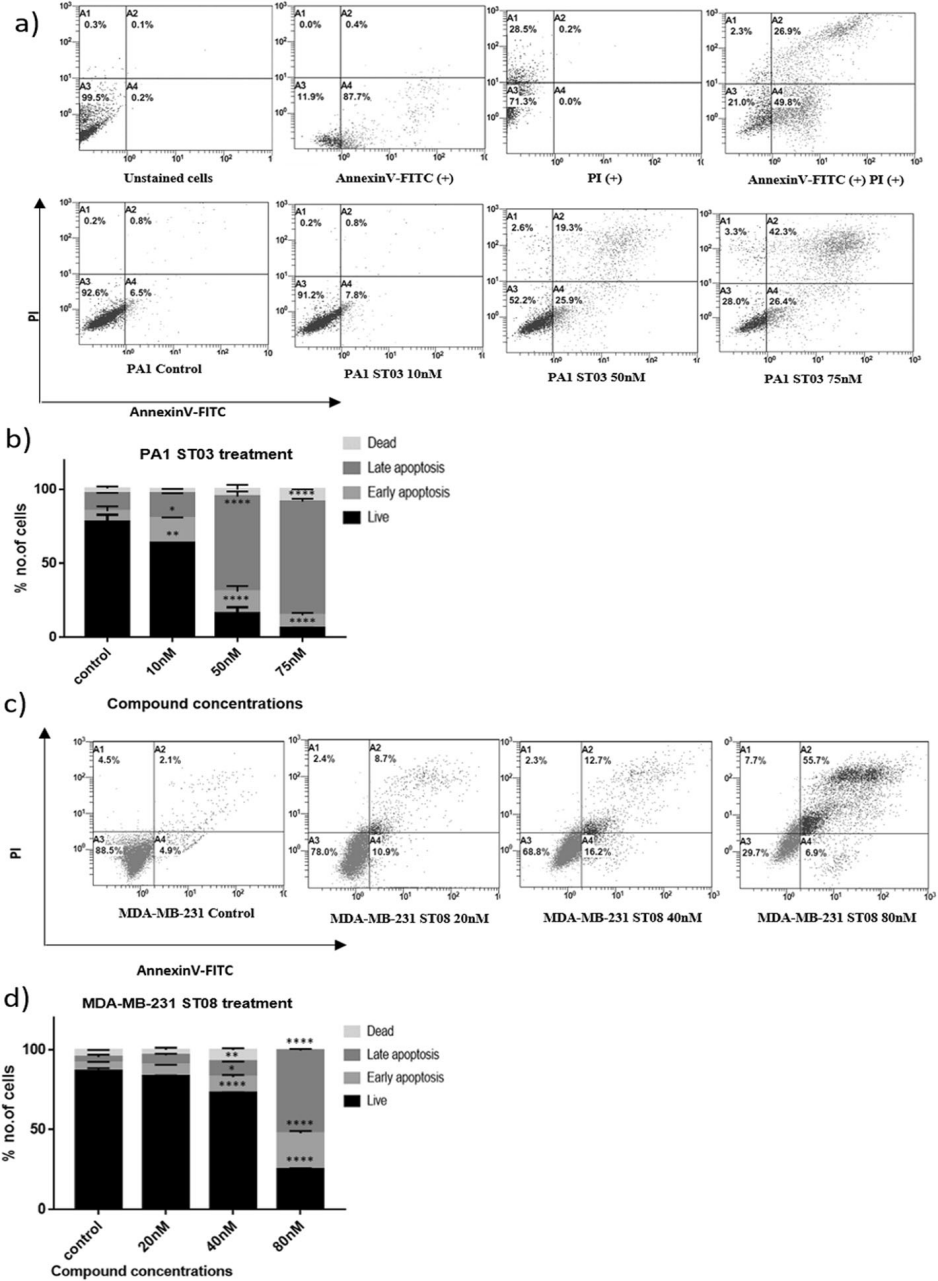
Evaluation of cell death by ST compound treatments using AnnexinV-FITC/PI. **a** ST03 treated PA1 cells stained with Annexin/FITC-PI and **b** Quantification of percentage of cells in each stage **c** ST08 treated MDA-MB-231 cells stained with Annexin/FITC-PI and **d)** Quantification of cells in each stage. Each experiment was repeated for a minimum of 3 times and plotted as bar graphs with error bars. Two-way ANOVA was conducted and the p value was calculated between control and ST03 treated groups, where, *: p value < 0.05, **: p value < 0.005, ***: p value < 0.0001, ****: p value < 0.00001

Since our novel compounds ST03 and ST08 inhibited the cancer cell proliferation, we were interested in analyzing the mechanism by which the compounds induce cytotoxicity on ovarian and breast cancer cell lines. It is well known that anti-cancer compounds can cause different types of cell deaths such as apoptosis, necrosis or autophagy, thereby determining the fate of cell [35]. Thus, to dig into the causative mechanism, ovarian and breast cancer cells were treated with ST03 and ST08, subjected to flow cytometry analysis after staining with annexin V-FITC-PI staining [36]. In live cells, PS (phosphatidylserine) component of the plasma membrane is facing the cytoplasmic side. In a cell undergoing apoptosis, PS flips towards the outer side. This can be recognised by Annexin V. In cells undergoing cell death via necrosis, PS is not flipped, but cells are leaky; therefore, PI enters the nuclei and stains DNA. Annexin V, together with Propidium iodide (PI) is used for the detection of apoptotic cell population based on the integrity of the plasma membrane. The dot blots obtained from flow cytometry analysis shown in Fig. 5 describes the population of cells at different stages of cell death. The lower left quadrant contains cells that are live, negative for both the stains (Annexin ^(−)^ PI ^(−)^), lower right quadrant is occupied by early apoptotic cells (Annexin ^(+)^ PI ^(−)^), upper right quadrant contains late apoptotic cells (Annexin ^(+)^ PI ^(+)^) and the upper left quadrant has necrotic/dead cells (Annexin ^(−)^ PI ^(+)^). The results shown in Fig. 5a-d depict that, both ST03 and ST08 induce a greater extent of apoptotic cell death in ovarian and breast cancer cell lines, respectively.

The representative dot plot shows that the 50 nM concentration of ST03 could induce 47.8% of cells death, in which 25.9% were early apoptotic (Annexin ^(+)^ PI ^(−)^), 19.3% were late apoptotic (Annexin ^(+)^ PI ^(+)^), 2.6% were necrotic (Annexin ^(−)^ PI ^(+)^) in PA1 (Fig. 5a, b). Maximal accumulation of early apoptotic population (16.2%) was seen at 40 nM of ST08 on breast cancer cell line MDA-MB-231. And at 80 nM, these early populations have migrated to the third compartment, leaving 29.7% unstained, 6.9% in early, 55.7% in late and 3.2% in necrotic stages (Fig. 5c, d). From these results, it is evident that both compounds exhibited cell death via apoptosis.

### ST03 and ST08 induce the intrinsic apoptotic pathway in breast and ovarian cancer cell lines

**Fig. 6:**
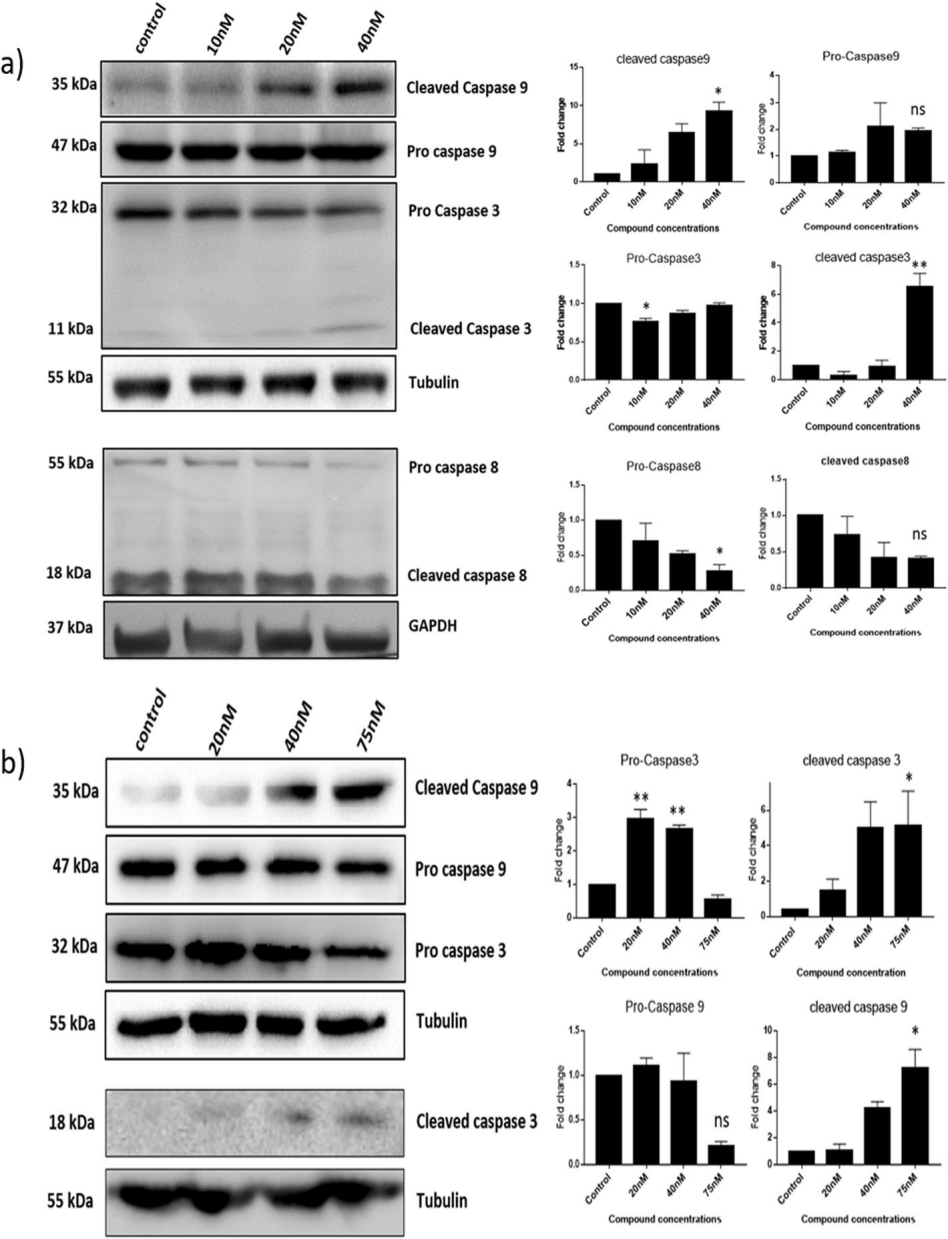
Expression of apoptotic proteins PA1 and MDA-MB-231 cells on compound treatment. **a** Western blot image of PA1 cell lysate treated with ST03 compound for 48 h **b** Western blot image of MDA-MB-231 cell lysate treated with ST08 compound for 48 h. Each experiment was repeated for a minimum of 3 times and plotted as bar graphs with error bars. One way ANOVA was conducted and the p value was calculated between control and ST03 treated groups, where, *: *p* value < 0.05, **: *p* value < 0.005

As we observed apoptotic populations in the treated cells from annexin V-FITC/PI assay results; we were curious to know the pathway of apoptosis. Thus, to further investigate the cell death pathway (intrinsic vs extrinsic pathway of apoptosis) induced by ST03 and ST08, we checked the expression of key proteins involved in apoptotic pathways using western blotting. Caspase-9 is an initiator caspase which is a part of mitochondria-mediated intrinsic apoptotic pathway and is activated by cytochrome-c released from mitochondria. At the same time, caspase-3, being the executioner caspase, is responsible for the proteolysis of α-fodrin, PARP, gelsolin, ICAD and other caspases leading to the effective completion of apoptosis process [37, 38]. In our treatments, both the compounds significantly increased the amount of cleaved/active caspase 9 and cleaved/ active caspase 3 in PA1 and MDA-MB-231 cancer cell lines Fig. 6a-b. On the other hand, the extrinsic apoptotic marker procaspase 8 and its active form were found to be downregulated. Both ST03 and ST08 induce the intrinsic apoptotic pathway in ovarian cancer cell line PA1 and in breast cancer cell line MDA-MB-231.

### ST08 inhibits breast cancer cell migration

**Fig. 7:**
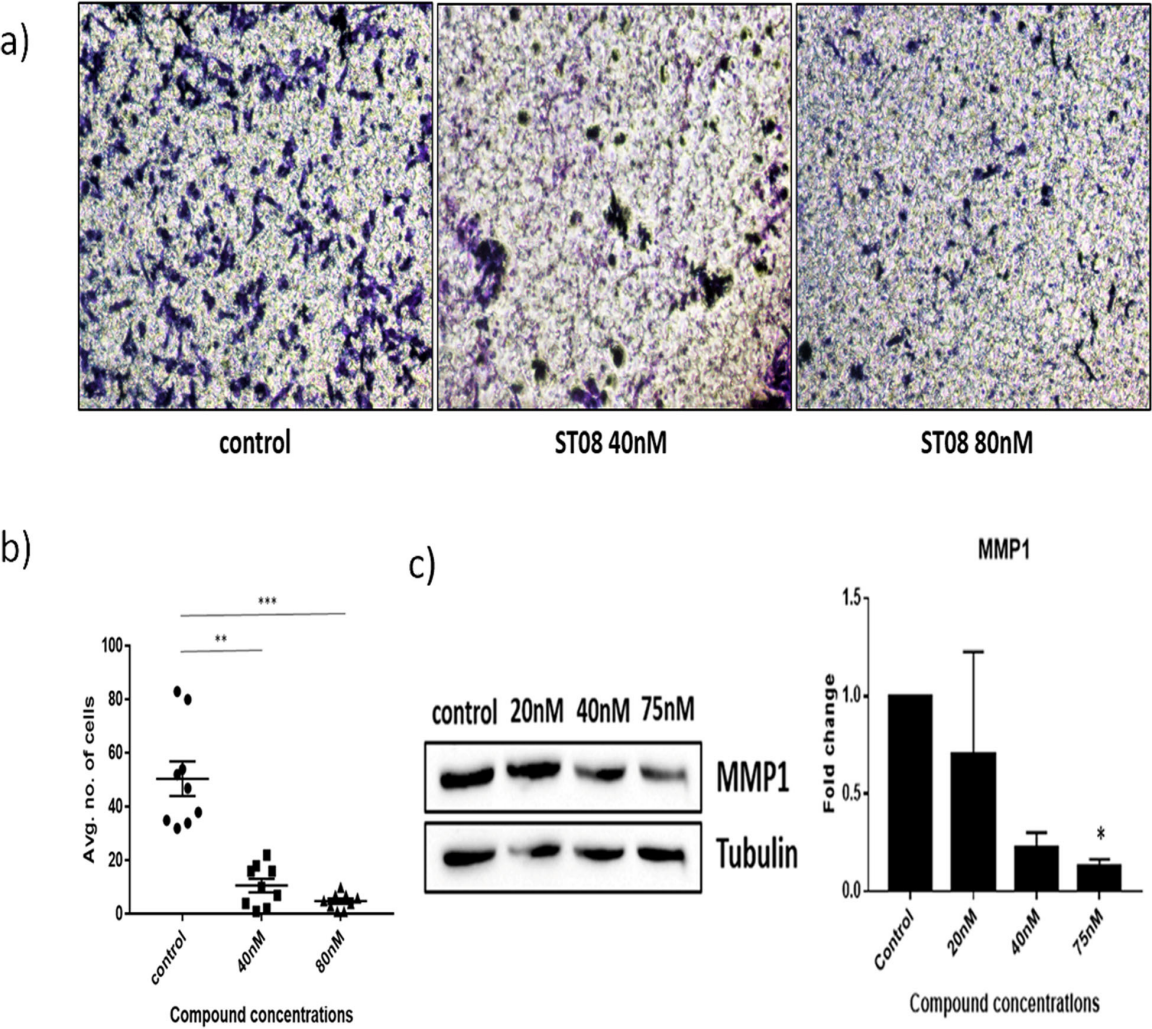
Effect of ST08 on MDA-MB-231 cell migration **a** ST08 treated MDA-MB-231 migration in vitro and **b** its quantification **c)** Expression of MMP1 in ST08 treated MDA-MB-231 cells**.** Each experiment was repeated for a minimum of 3 times and plotted as bar graphs with error bars. One-way ANOVA was conducted and the p value was calculated between control and ST03 treated groups, where, *: *p* value < 0.05, **: *p* value < 0.005 *: *p* value < 0.05, **: *p* value < 0.005, ***: *p* value < 0.0001, ****: *p* value < 0.00001

The effect of ST08 on the migratory ability of MDA-MB-231 was evaluated by Transwell migration assay. Compared with untreated control cells, the migration capacity of the 24 h treated cells was significantly diminished (Fig. 7a, b). The migration inhibition rate was 3 fold in the treated cells (Fig. 7b) when compared to the untreated control groups. Besides, we examined the expression of MMP1 (Fig. 7c) in the ST08 treated MDA-MB-231 cells. Interestingly, MMP1 showed reduced expression at higher concentrations (80 nM) of ST08. Cell migration is the most important event that happens as an initial step of metastasis [39]. This result indicates that ST08 can inhibit the cancer cell migration effectively, which can prevent metastasis and progression of cancer.

## Discussion

Drug toxicity and disease relapse remain as major hurdles in the management of cancer despite having advanced targeted therapies. Plant-derived low toxic phenolic compounds are known to exhibit anti-cancer activity by inducing apoptosis in cancer cells [27, 40, 41]. Curcumin is one such example which exhibits anti-cancer activity against various cancer types [42–47]. Curcumin has been replaced by more potent and bioavailable derivatives, which have attracted attention for the development of novel drugs [48]. The novel curcumin derivatives developed in this study has exhibited remarkable cytotoxicity (~ 100X) better than parent compound at nanomolar concentrations on a wide range of cancer cell lines which includes both representative liquid and solid tumour cell lines. Various types of curcumin derivatives have been reported, to overcome the low potency, poor bioavailability and side effects demonstrated by the parent compound curcumin [49]. One among them is 1,2-bis[(3E,5E)-3,5-dibenzylidene-4-oxo-1-piperidyl]ethane-1,2-diones, which exhibited anti-cancer activity on variety of cancer cell lines [27–29]. Our novel compounds ST08 and ST03 synthesised from 1,2-bis[(3E,5E)-3,5-dibenzylidene-4-oxo-1-piperidyl]ethane-1,2-dione, by adding strong electron-withdrawing groups such as NO_2_ and Cl- respectively to the backbone, has enhanced the anti-proliferative effect on the cancer cells particularly on ovarian cells (PA1) with stem cell-like properties. The cytotoxicity assays performed showed inhibition of cell growth by ST03 and ST08 in the range of 30–125 nM in both adherent and non- adherent cell lines which is ~ 100 fold better than the parent compound curcumin, which was effective in the range of ~ 10–100 μM on most of the cancer cells lines reported till date [43, 44, 50]. ST03 and ST08 are stable and bioavailable better than Curcumin (data not shown).

Further analysis showed that ST03 was consistent in its effect among all cell lines tested with an average IC_50_ value of ~ 36 nM. Among the ovarian cancer cell lines, PA-1 (teratocarcinoma, undifferentiated) and A2780 (epithelial cancer cell line, differentiated), ST03 showed the better effect on PA1 cells (41 nM) as compared to A2780 (54 nM). Similarly, ST08 was found to show differential cytotoxicity with PA1 (38 nM) better than A2780 (64 nM). Since ovarian cancer cell lines showed promising results, we checked the effect of ST08 on breast cancer cell lines. We took MDA-MB231 (triple negative, basal, metastatic) and MCF-7 (epithelial, luminal) for the comparison. As observed in the ovarian cancer cell line, the highly metastatic MDA-MB231 showed better cytotoxicity (54 nM) than MCF-7 (127 nM). It is to be noted that, ST03 and ST08 showed cytotoxicity on undifferentiated cancer cells populations which have the potency to migrate (metastatic) and differentiate (stem cell characteristics). A comparison of the IC_50_ value of both the ST compounds has revealed the peculiar characteristics of them on the MDA-MB-231 and PA1 cell lines tested. It showed that these novel compounds are highly potent on PA1 and MDA-MB-231 cell lines (Table 1 and 2), which possess some of the properties of undifferentiated cells [51–54].

PA1 belongs to the category of teratocarcinoma, where the tumour cells have originated from germLine cells which have the characteristics of stem cells [52]. For instance, although we observe reasonable response rates to first-line chemotherapy, recurrence with resistance occurs in most of the cases of ovarian cancer, which is delivered by cancer stem cells that remain even after treatment [55, 56]. It is known that cancer stem cells expansion and characteristics lead to drug resistance [57, 58]. For the improved treatment outcome without relapse, it is necessary to eliminate cancer stem cells. The new treatment strategies of ovarian cancer are thus looking for drugs that can remove cancer stem cells very effectively. Drugs such as AS602801, and 673A has proved to eradicate ovarian cancer stem cells, and it increased the sensitivity of cancer stem cells to standard drugs such as cisplatin and paclitaxel [59, 60]. When compared to the potency of these drugs, ST03 showed cytotoxicity at a nanomolar concentration in PA1 cells (undifferentiated stem cell-like) in vitro. Thus the likelihood of improvement in anti-cancer activity cannot be ruled out if ST03 and ST08 are used in combination with standard of care drugs. Owing to the fact that ovarian cancer stem cells are relatively challenging to eradicate, the observed cytotoxicity of ST03 on PA1 is promising.

In the case of epithelial ovarian cancer cell line A2780, the effect of ST compounds were observed at a little higher concentration than on PA1. This observation is crucial as it indicates the differential cytotoxicity of ST compounds on tumors of different origin. Differential sensitivity of cell lines to ST compounds would help in understanding drug metabolism in cell types and thereby help to combat the acquired resistance. This knowledge would ultimately help in deciding combination therapies [61] .

Lack of multiple receptors is the major characteristics of the triple-negative breast cancer cell line MDA-MB-231 [62] and these cells respond the least to hormone therapies [63]. As a reason, potent inhibitors derived from natural compounds that can kill triple-negative breast cancer cells were developed in our laboratory. The effectiveness was predominant on MDA-MB-231, the triple-negative breast (highly metastatic) cancer cell line [39, 64–66] than on the non-metastatic cell line MCF7. This result is very promising since the triple-negative breast cancer types are highly metastatic and difficult to treat, as there are a fewer treatment strategy for triple-negative breast cancer types [67].

To study the selectivity of ST03 and ST08 between cancer and normal, peripheral blood mononuclear cells and 293 T cell lines were used. It was found that ST03 (> 150 nM) and ST08 (> 150 nM) are not cytotoxic to them at concentrations, at which it was cytotoxic to the non- adherent cancer cell lines. This points onto a fascinating fact that, these compounds are selective towards cancer cells and are less effective on normal cells which would render less systemic toxicity.

Metastasis is the primary cause of high mortality rate seen in cancer patients [68] which justifies the requirement of drugs with migrastatic potential. Previous reports have established the migrastatic property of curcumin [69] in cancer cells. Metastatic cell types undergo EMT (Epithelial to mesenchymal transition) during metastasis [70]. EMT also occurs not only in metastasis but also during wound healing and helps in tissue regeneration [70]. Recently, reports have linked EMT and dedifferentiated cancer stem cell-like properties of cancer cells. This points out the fact that cancer cells when they undergo EMT transition, it also acquire cancer stem cell-like features which initiates metastasis [71, 72]. The anti-cancer agent which targets the metastatic cancer cells can restrain the cancer stem cell-like cells or vice versa. Our study has demonstrated the cytotoxic effect of ST compounds on both liquid and solid cancer types, including stem cell-like PA1 and highly metastatic MDA-MB-231. This observation made us hypothesise the anti-metastatic potential of ST compounds.

Interestingly, we observed anti-metastatic property of ST08 compound on the highly aggressive, triple-negative, metastatic, MDA-MB-231 cell line. There was 4–5 fold reduction observed in migration of the MDA-MB-231 cells upon ST08 treatment. In addition, MMP1, the matrix metalloprotease reported to be one of the key players in breast cancer cell metastasis [73], was also found to be downregulated on ST08 treatment. Our results are in agreement with the characteristics of parent compound curcumin which also can block metastasis of cancer cells. Hence, our results report for the first time that ST compounds can suppress cancer cells possessing metastatic and cancer stem cell-like properties in vitro. These characteristics of our compounds might, therefore, open up a new strategy for treating recurrent malignant cancer types.

For the elimination of old cells and the growth of new cells, tightly regulated apoptosis process is required, and it plays a vital role in the growth and development of an organism [74]. Deregulation of apoptosis in a group of cells leads to proliferation of cells, cancer. Anti-cancer drugs such as platinum compounds and taxanes usually induce apoptosis in cancer cells [75]. Here in our study, we observed early and late apoptotic events in both the treatments. Further, there are majorly two types of apoptosis, mitochondrial-mediated intrinsic and death receptor-mediated extrinsic [76, 77]. Our results demonstrated that both the compounds are inducing cell death via intrinsic apoptosis as there is an upregulation in the expression of caspases such as caspase 3 and caspase-9. Caspases such as caspase-9 and caspase-3 are predominant proteins involved in the process of the intrinsic apoptotic pathway. A cascade activation of these molecules results in activation of PARP, cleavage of cytoskeleton proteins and chromatin fragmentation leading to apoptosis [78, 79]. We observed an increase in the expression of a cleaved form of caspase-9 in both the cell lines treated with ST03 or ST08. In the PA1 cell line, ST03 treatment has increased the expression of cleaved caspase-3 and caspase-9 indicative of the intrinsic pathway of apoptosis. Similarly increase in cleaved caspase-9 and caspase-3 was indicative of intrinsic pathway of apoptosis in ST08 treated MDA-MB-231.

The in vitro experiments conducted here, demonstrate the effective anti-cancer property of our novel curcumin derivatives ST03 and ST08. The compounds were found to be ~ 100 fold more potent than its parent compound, curcumin on both solid and liquid tumour types. The fascinating fact here is that they exhibited higher toxicity on cancer cell lines such as PA1 and MDA-MB-231, which possess cancer stem cell-like property to differentiate and migrate. The reduced expression of MMP1 and inhibition of migration on ST08 treatment indicate that this compound can modulate metastasis by inhibiting migration of cancer cells. Eradication of cancer stem cells and inhibition of metastasis could effectively reduce cancer recurrence and drug resistance which is a major hurdle in the cancer treatment. Hence, our results suggest that ST03 and ST08 can be considered as a promising candidate anticancer drug that can target both cancer stem cells and metastatic cells mostly responsible for the failure of cancer therapy. Further studies are required to understand the mechanism of cytotoxicity of these compounds, which is in progress.

## Conclusion

We report for the first time two novel compounds, ST03 and ST08, which exhibit anti-cancer activity on both liquid and solid cancer types. These are ~ 100 fold better than its parent compound curcumin. We also show that these compounds inhibit cell proliferation by inducing intrinsic apoptosis and also suppressing cell migration in vitro. Due to the anti-cancer and migrastatic effect of ST03 and ST08 on stem cell-like ovary and breast cancer cell lines. These compounds have the potential to be developed as a novel anticancer agent towards the treatment of the metastatic, invasive, and recurrent cancer types.

## Supplementary information

**Additional file 1: Table S1.** Curcumin structure and IC50 values. **Figure S1.** Mass spectrometry data. **Figure S2.** ^1^HNMR data.

## Data Availability

Raw data used in this study and presenting tables and figures is sufficient to state that all data is contained within the manuscript and additional files.

## Abbreviations

CDCl3: Deuterated chloroform
DAP: Diarylidenyl-piperidone
DMSO: Dimethyl sulphoxide
ELISA: Enzyme-linked immunosorbent assay
FITC/PI: Fluorescein isothiocyanate/Propidium iodide
IR: Infrared
LDH: Lactate dehydrogenase
MP: Melting Point
MS (ESI): Mass spec (Electron Spray Ionisation)
MTT: 3-(4,5-dimethylthiazol-2-yl)-2,5-diphenyltetrazolium bromide
NMR: Nuclear Magnetic resonance
Rf: Retardation factor
