# Supplementary material for: Investigation of anti-cancer and migrastatic properties of novel curcumin derivatives on breast and ovarian cancer cell lines
Source: BMC Complement Altern Med. 2019 Oct 21;19:273. doi: 10.1186/s12906-019-2685-3 (PMC6802352; doi:10.1186/s12906-019-2685-3)
Supplement: Supplementary file 1 — Additional file 1: Table S1. Curcumin structure and IC50 values. Figure S1. Mass spectrometry data. Figure S2. 1HNMR data. [file 12906_2019_2685_MOESM1_ESM.docx]

**Investigation of anti-cancer and migrastatic properties of novel curcumin derivatives on breast and ovarian cancer cell lines**

Jinsha Koroth^a,b^, Snehal Nirgude^a,b^, Shweta Tiwari^d^, Vidya Gopalakrishnan^a,b,c^, Raghunandan Mahadeva^a^, Sujeet Kumar^d^, Subhas S Karki^d^, ^*^Bibha Choudhary^a^

*a. Institute of Bioinformatics and Applied Biotechnology, Electronic city phase 1, Bangalore, 560100, India*

*b. JK , SN and VG are graduate students registered under Manipal Academy of Higher Education, Manipal, 576104, India*

*c. Department of Biochemistry, Indian Institute of Science, Bangalore,560012, India*

*d. Department of Pharmaceutical Chemistry, KLE Academy of Higher Education & Research, KLE College of Pharmacy, Rajajinagar, Bangalore, KN, India*

*Corresponding author :

Bibha Choudhary

Institute of Bioinformatics and Applied Biotechnology

Electronic city phase 1, Bangalore, Karnataka, India

Tel.:080-285 289 00, fax: 080-285 289 04

e-mail: vibhachou@gmail.com

**Supplementary information:**

**Results:**

| **Curcumin** | |
| --- | --- |
| 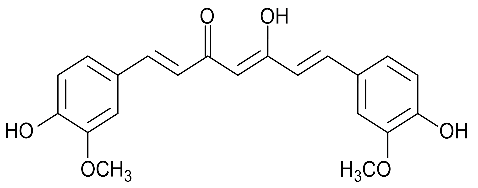 | |
| **Cell lines** | **IC50 (uM)** |
| Nalm6 | 12 |
| K562 | 0.056 |
| PA1 | 6 |
| A2780 | 7 |

**Table S1: Curcumin structure and IC50 values**

**Figure S1: Mass spectrometry data:**

**ST03:**

**
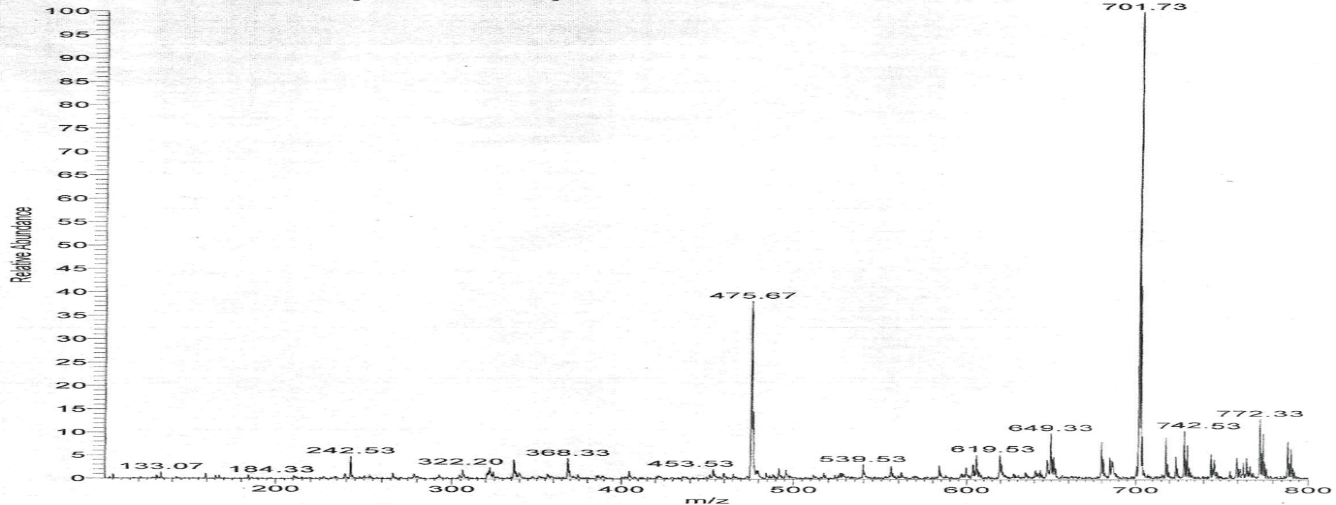
**

**ST08:**

**
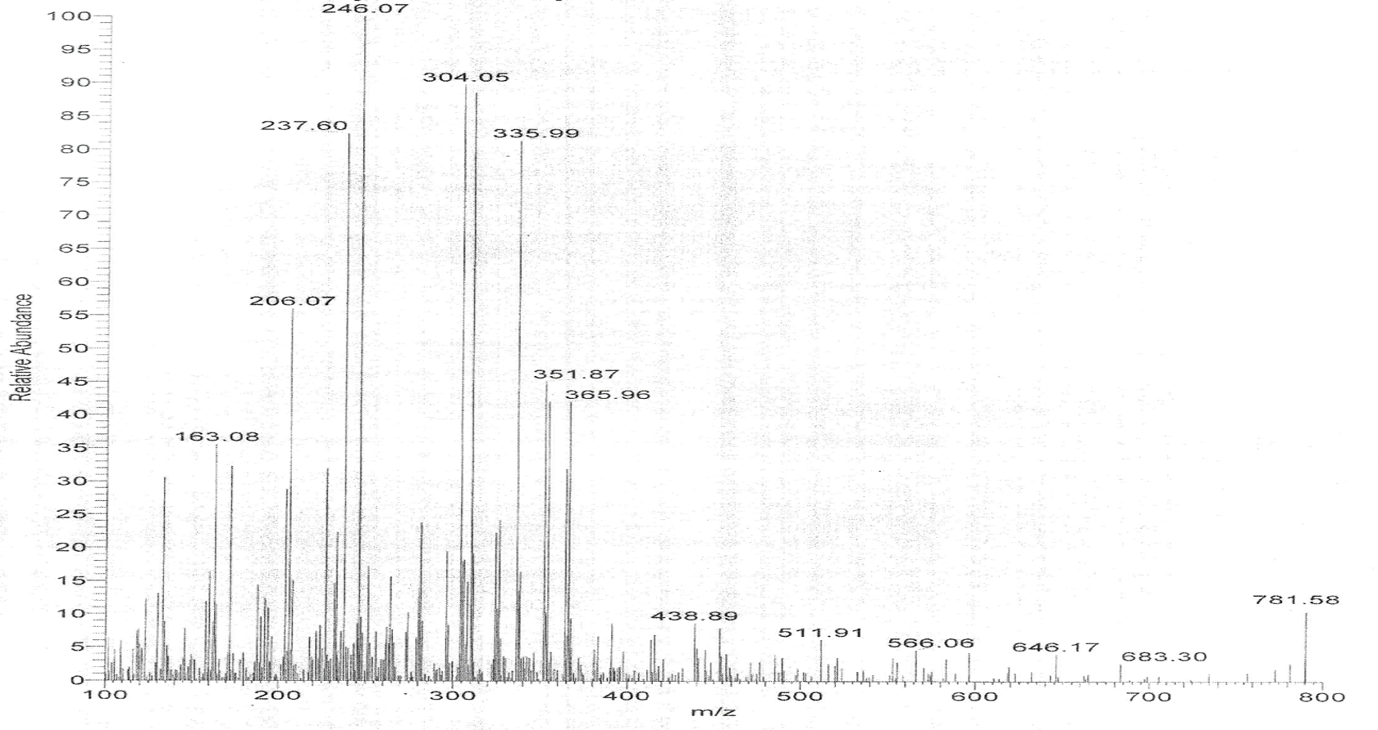
**

**Figure S2: ^1^HNMR data:**

**ST03:**

**
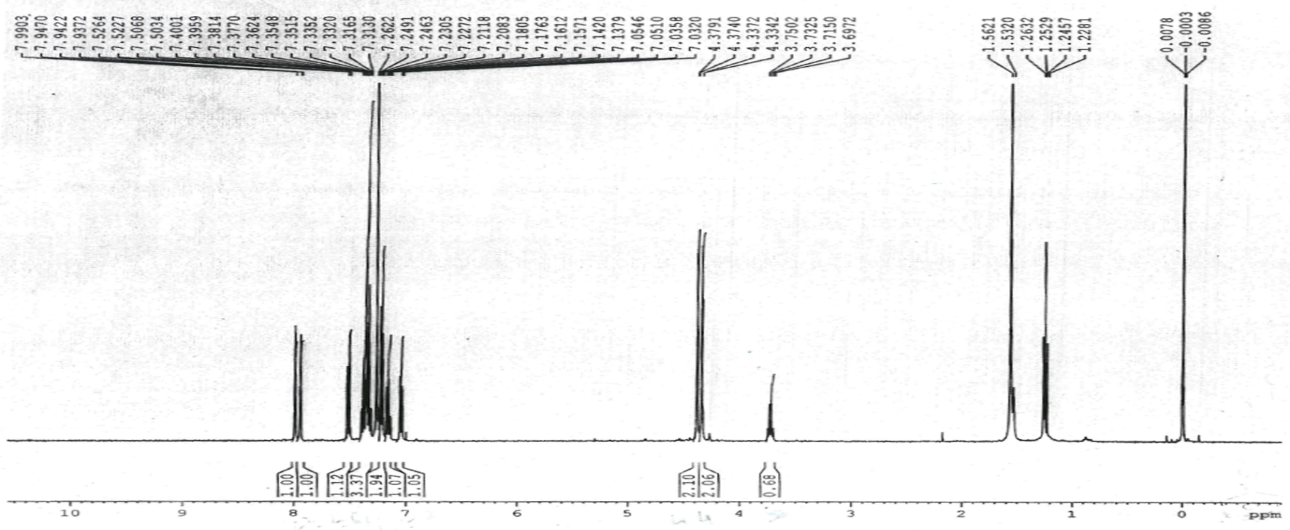
**

**ST08:**

**
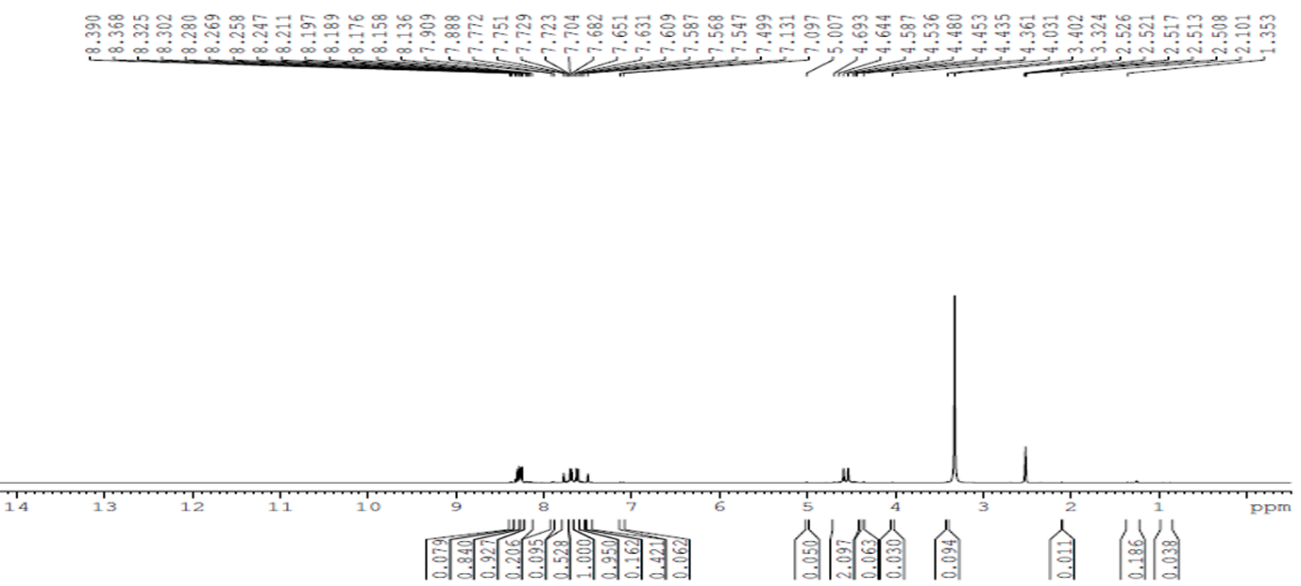
**
